# Sinusoidal CO_2_ respiratory challenge for concurrent perfusion and cerebrovascular reactivity MRI

**DOI:** 10.3389/fphys.2023.1102983

**Published:** 2023-02-09

**Authors:** Chau Vu, Botian Xu, Clio González-Zacarías, Jian Shen, Koen P. A. Baas, Soyoung Choi, Aart J. Nederveen, John C. Wood

**Affiliations:** ^1^ Department of Biomedical Engineering, University of Southern California, Los Angeles, CA, United States; ^2^ Division of Cardiology, Children’s Hospital Los Angeles, University of Southern California, Los Angeles, CA, United States; ^3^ Neuroscience Graduate Program, University of Southern California, Los Angeles, CA, United States; ^4^ Signal and Image Processing Institute, University of Southern California, Los Angeles, CA, United States; ^5^ Department of Radiology and Nuclear Medicine, Amsterdam UMC, Location AMC, Amsterdam, Netherlands

**Keywords:** brain perfusion, respiratory challenges, cerebrovascular reactivity (CVR), carbon dioxide challenge, deoxygenation, dynamic susceptibility contrast (DSC)

## Abstract

**Introduction:** Deoxygenation-based dynamic susceptibility contrast (dDSC) has previously leveraged respiratory challenges to modulate blood oxygen content as an endogenous source of contrast alternative to gadolinium injection in perfusion-weighted MRI. This work proposed the use of sinusoidal modulation of end-tidal CO_2_ pressures (*SineCO*_*2*_), which has previously been used to measure cerebrovascular reactivity, to induce susceptibility-weighted gradient-echo signal loss to measure brain perfusion.

**Methods:** *SineCO*_*2*_ was performed in 10 healthy volunteers (age 37 ± 11, 60% female), and tracer kinetics model was applied in the frequency domain to calculate cerebral blood flow, cerebral blood volume, mean transit time, and temporal delay. These perfusion estimates were compared against reference techniques, including gadolinium-based DSC, arterial spin labeling, and phase contrast.

**Results:** Our results showed regional agreement between *SineCO*_*2*_ and the clinical comparators. *SineCO*_*2*_ was able to generate robust CVR maps in conjunction to baseline perfusion estimates.

**Discussion:** Overall, this work demonstrated feasibility of using sinusoidal CO_2_ respiratory paradigm to simultaneously acquire both cerebral perfusion and cerebrovascular reactivity maps in one imaging sequence.

## 1 Introduction

Perfusion magnetic resonance imaging (MRI) is a popular imaging technique for assessing hemodynamic impairments in a variety of central nervous system abnormalities such as intracranial tumors and acute strokes (Jahng et al., 2014). There are multiple different MRI techniques to measure cerebral perfusion, including phase contrast (PC), arterial spin labeling (ASL), and dynamic susceptibility contrast (DSC).

Particularly, DSC MRI is a perfusion technique that is frequently performed in clinical routines, requiring intravenous injection of a contrast agent (gadolinium chelate) and dynamic imaging to capture the passage of the contrast bolus through the vasculature (Østergaard, 2005). Based on the susceptibility-induced signal loss caused by the paramagnetic contrast, tracer kinetics models are applied to calculate multiple perfusion parameters. Despite its popular usage and clinical utility, DSC suffers from its reliance on exogenous gadolinium contrasts, which pose increased risks of anaphylaxis, nephrogenic systemic fibrosis, (Schlaudecker and Bernheisel, 2009) and gadolinium deposition in different tissues (Strickler and Clark, 2021).

To address this drawback, recent works have proposed contrast-free deoxygenation-based DSC (dDSC) which take advantage of endogenous paramagnetic deoxyhemoglobin to induce susceptibility-weighted MRI signal losses, similar to the effects of gadolinium (MacDonald et al., 2018; Poublanc et al., 2021; Vu et al., 2021). This dDSC technique delivers boluses of deoxygenated hemoglobin through transient exposure to low-oxygen (hypoxia) or high-oxygen (hyperoxia) gas inhalation (Meier and Zierler, 1954; Østergaard et al., 1996; Østergaard, 2005) and has demonstrated feasibility in healthy volunteers as well as chronic anemia subjects who had elevated blood flow and shortened transit time (Vu et al., 2021).

**FIGURE 1:**
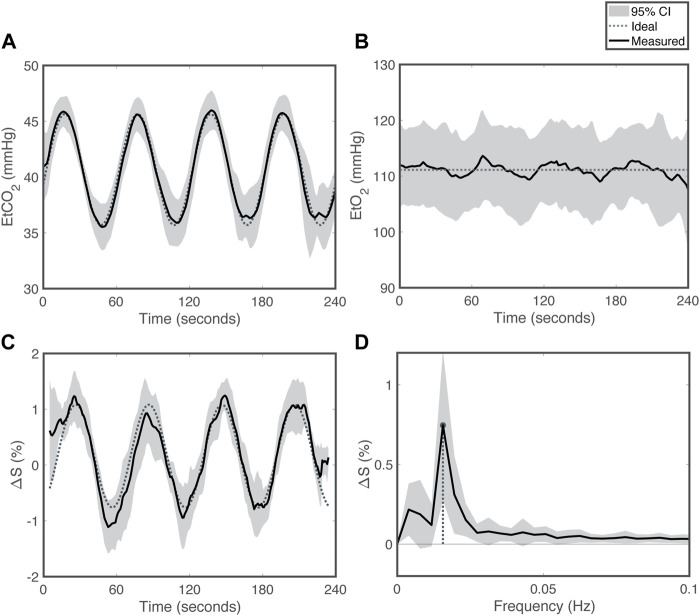
Respiratory challenge patterns for *SineCO*_*2*_ **(A)** End-tidal carbon dioxide (EtCO_2_), **(B)** end-tidal oxygen (EtO_2_), **(C)** percent signal change in the time domain and **(D)** in the frequency domain. Grey shading reflects 95% confidence interval. Dotted line represents the targeted sequence, and solid line is the average time series measured in the cohort.

One of the obstacles to perfusion quantification in both gadolinium-based and deoxygenation-based DSC is the determination of cerebral blood flow (CBF), which requires a deconvolution between the signals in the blood and in the tissue. Traditionally, this deconvolution is performed using a singular value decomposition (SVD) approach in the time domain (Østergaard et al., 1996). In this work, we propose to replace the transient contrast bolus with a sinusoidal gas challenge and compute perfusion at the fundamental sinusoidal frequency in the Fourier domain, thereby simplifying the SVD deconvolution process. We also propose to raise and lower the concentration of deoxygenated hemoglobin through modulations of end-tidal CO_2_ level (Figure 1A), rather than manipulating the inspired oxygen concentration. The sinusoidal end-tidal CO_2_ fluctuations (*SineCO*_*2*_), and corresponding changes in oxygen delivery, trigger reciprocal changes in the gradient-echo MRI signals that can be converted into CBF estimates. In order to assess the feasibility of this new perfusion technique, we evaluated *SineCO*_*2*_ on 10 healthy volunteers in comparison with perfusion measurements from standard gadolinium-based DSC, ASL, and PC MRI.

## 2 Materials and methods

### 2.1 Study protocol

The Committee on Clinical Investigation at Children’s Hospital Los Angeles approved the protocol; written informed consent was obtained from all subjects (CCI#20-00050). This study was performed in accordance with the Declaration of Helsinki.

A total of 10 healthy volunteers participated in this study during the months of April and May of 2021. Exclusion criteria included pregnancy, hypertension, diabetes, stroke or other known neurologic insult, seizures, known developmental delay or learning disability, at least one ‘yes’ answer to the 6-question Choyke survey (Choyke et al., 1998), and measured glomerular filtration rate (GFR) lower than 60 mL/min/1.73 mm^2^ (Østergaard, 2005). Imaging, vital signs (heart rate, blood pressure, temperature, and oxygen saturation), and blood samples (for complete blood count) were collected for each subject on the same study visit date.

### 2.2 Respiratory challenges

Respiratory challenges were performed by prospectively targeting end-tidal O_2_ (EtO_2_) and end-tidal CO_2_ (EtCO_2_) partial pressures using a specialized computer-controlled gas blender (RespirAct, Thornhill Research, Toronto, Canada) (Slessarev et al., 2007). This device measures the subject’s baseline EtO_2_ and EtCO_2_ during the initial preparation phase and delivers specific concentrations of oxygen and carbon-dioxide during the challenge phase to accurately target EtO_2_ and EtCO_2_ values. Fingertip pulse oximetry SpO_2_ (Nonin, Plymouth, MN) was recorded continuously during gas challenges. *SineCO*_*2*_ challenge was performed, in which EtO_2_ was clamped at subject-specific baseline and EtCO_2_ was modulated in a sine wave between 35 and 45 mmHg with a period of 60 s (Blockley et al., 2011). This period was fourfold longer than the brain’s characteristic rise-time in response to CO_2_ (Duffin et al., 2015) and has previously been used in published CVR protocols (Blockley et al., 2011).

### 2.3 MRI experiment

#### 2.3.1 Structural MRI

All MRI was acquired on a 3T Philips Achieva (Philips Medical Systems, Best, Netherlands) with a 32-channel head-coil. Pre-contrast anatomical 3D T1 was acquired with the following parameters: TR = 8 ms, TE = 3.7 ms, flip angle = 8°, and resolution = 1 mm isotropic. Total scan time was 5:18 for T1 sequence.

Pre-processing steps on structural T1-weighted images consist of brain extraction, tissue classification into grey matter (GM), white matter (WM) and segmentation using the BrainSuite Anatomical Pipeline (brainsuite.org, v.21a). Tissue segmentation into 312 regions-of-interest (ROI) was performed using the USCBrain anatomical atlas (Joshi et al., 2022), whose labels were modified to include subdelineations of deep WM tissue (manually drawn WM structures) and the cerebellum [transferred from the probabilistic atlas of the human cerebellum (Diedrichsen et al., 2009)]. All cortical ROIs were separately labeled into GM and gyral WM regions. Subsequently, these labels were transferred to each subject’s structural imaging space.

#### 2.3.2 Arterial spin labeling (ASL)

Time-encoded pseudo-continuous ASL was acquired with the following parameters: TE = 16 ms, TR = 5,040 ms, Hadamard-8 matrix with seven blocks of 2,000, 800, 500, 300, 250, 200, and 150 ms, post-label delay (PLD) = 100 ms, SENSE = 2.5, resolution = 3 mm × 3 mm × 6 mm, FOV = 240 mm × 240 mm × 114 mm, two FOCI background suppression pulses, 2D single-shot EPI readout, and 12 signal averages. M0 images were acquired by switching off labeling and background suppression and keeping the same imaging parameters, except for TR = 2,500 ms. Scan time was 8:44 for ASL sequence and 18 s for M0 sequence.

Perfusion quantification was performed using FSL BASIL toolbox (FSL, Oxford, United Kingdom). Additional details on acquisition and processing of the time-encoded ASL sequence have been previously published (Afzali-Hashemi et al., 2021). Briefly, all perfusion weighted images were motion-corrected to the first dynamic using SPM12 (Wellcome Trust Center for Neuroimaging, London, United Kingdom). The individual acquisitions were subsequently subtracted according to a Hadamard-8 matrix to obtain perfusion weight images having PLD values of 100, 250, 450, 700, 1,000, 1,500, and 2,300 ms. The individual PLD images experienced different numbers of background suppression pulses and were divided by a correction factor of (0.95)^N^, where N was the number of pulses. The signal variation across PLD was denoised using a spatiotemporal generalized variation model as described by (Spann et al., 2017). The denoised perfusion weighted images were processed using the BASIL toolbox which uses Bayesian inference to fit arterial transit time, arterial blood volume and CBF voxelwise using an extended kinetic model (Chappell et al., 2010). Blood T1 was derived from the subject’s measured hematocrit (Lu et al., 2004). Subject-specific labeling efficiency was derived from the flow-weighted velocity measured from the phase contrast images (Aslan et al., 2010). The final maps were smoothed using a Gaussian lowpass filter with full-width half maximum value of 3.5 mm.

Subsequently, perfusion maps were registered to dDSC native space for comparison.

#### 2.3.3 Phase contrast (PC)

Single-slice PC images were acquired above the carotid bifurcation: TR = 17 ms, TE = 10 ms, flip angle = 10°, resolution = 0.6 mm × 0.6 mm, FOV = 220 mm × 220 mm, slice thickness = 5 mm, and velocity encoding gradient of 80 cm/s. The scan was ungated and used 10 averages to compensate for pulsatility. Scan time was 1:06 for the phase contrast acquisition. Details on calculation of total CBF from four feeding arteries were published in previous works. (Coloigner et al., 2020; Wymer et al., 2020). Briefly, vessel edges were derived from the complex-difference images using Canny edge detection from MATLAB (MathWorks, Natick, MA). Subsequent vessel areas were mapped to the phase-difference (velocity) images and total brain blood flow was calculated as follows:

$$\overset{4}{\underset{i=1}{\sum }}\overset{N}{\underset{j=1}{\sum }}V\left(j\right)$$ (1)

where V is the velocity map (after appropriate scaling for geometry and velocity encoding), the inner summation is across the N voxels in the vessel and the outer summation is across the four feeding vessels. The total CBF was then normalized to brain weight by calculating the total brain volume from the 3D T1w image (BrainSuite, brainsuite.org, v.21a) and assuming a brain density of 1.05 g/mL.

#### 2.3.4 Gadolinium-based DSC

Traditional gadolinium DSC was acquired using a dual-echo gradient-echo blood-oxygen level dependent (BOLD) MRI sequence with the following parameters: TR = 1.5 s, TE = 8/35 ms, flip angle = 30°, FOV = 190 mm × 190 mm × 100 mm, resolution = 2.5 mm× 2.5 mm × 5 mm, 160 dynamics, SENSE = 2, and no multi-band acceleration. The FOV was aligned with the previous dDSC acquisition at the time of scanning. Scan time was 4:05.

Gadovist at 0.1 mmol/kg was injected at a rate of 4 cc per second using a 20 or 22 gauge IV. Due to the lack of a power injector at our research facility, gadolinium contrast was injected manually by a physician. Contrast bolus was followed by 20 mL of saline flush *via* a three-way stopcock.

Gadolinium-based DSC BOLD images were preprocessed using the spatial functional processing pipeline similar to dDSC preprocessing, as detailed below. Perfusion values for CBF, cerebral blood volume (CBV), and mean transit time (MTT) were calculated based on published pipelines for gadolinium DSC (Stokes et al., 2021). Final rigid registration of perfusion images to deoxygenation DSC native space was performed for regional comparison.

#### 2.3.5 Deoxygenation-based DSC MRI

Dynamic gradient-echo BOLD MRI was acquired for the *SineCO*_*2*_ challenge with the following parameters: TR = 1.5 s, TE = 35/90 ms, flip angle = 52°, FOV = 190 mm × 190 mm × 100 mm, resolution = 2.5 mm isotropic, SENSE = 1, multi-band SENSE = 4, phase-encoding direction = AP, fat-shift direction = P, and 220 dynamics. One dynamic of reverse-gradient BOLD was acquired with the opposite fat-shift direction = A along the phase encoding direction. Scan time was 5:05 for the BOLD sequence and 9 s for the reverse-gradient BOLD.

To correct for EPI-induced distortion, BOLD images were pre-processed with field map calculated from opposite phase encoding directions. Motion correction with Analysis of Functional NeuroImages (AFNI, USA) and slice timing correction with FMRIB Software Library (FSL, Oxford, United Kingdom) were performed in that order. Registration of BOLD to T1 space was performed in BrainSuite. Finally, BOLD images were smoothed using a 4 mm × 4 mm × 4 mm Gaussian kernel.

All subsequent dDSC processing was performed in MATLAB (MathWorks, Natick, MA). Signal contribution from pial veins was suppressed by eliminating voxels with higher signal amplitude than the 98th percentile (Bhogal et al., 2022). Whole brain (WB), GM, and WM perfusion values was computed by averaging voxels within brain and tissue-specific masks in each subject’s functional native space.

### 2.4 Data analysis for deoxygenation-based DSC

#### 2.4.1 Gradient-echo $∆R_{2}^{*}$

Perfusion measures were calculated for both the single-echo data at 35 ms and dual-echo data at TEs of 35 and 90 ms. Single echo $∆R_{2}^{*}$ was calculated using the following relationship:

$$∆R_{2}^{*}\left(t\right)=-\frac{1}{TE}\ln\left(\frac{S\left(t\right)}{S_{0}}\right)$$ (2)

where $S\left(t\right)$ is the tissue signal and $S_{0}$ is the average value across $S\left(t\right)$ .

Dual echo ${∆R}_{2}^{*}$ was calculated for two echoes, TE_1_ = 35 ms and TE_2_ = 90 ms:

$${∆R}_{2}^{*}=\frac{1}{{TE}_{2}-{TE}_{1}}\left[\ln\left(\frac{S_{TE1}\left(t\right)}{S_{0,TE1}}\right)-\ln\left(\frac{S_{TE2}\left(t\right)}{S_{0,TE2}}\right)\right]$$ (3)

#### 2.4.2 Venous output function (VOF)

Venules have the largest BOLD fluctuation in response to CO_2_ stimuli because the blood volume is close to 100%, instead of <10% for brain tissue. The great cerebral veins not only have the largest signal intensity changes, but they have the longest delay relative to the global BOLD signal. Individual VOFs were obtained automatically by choosing 20 voxels with the highest integrated, rectified signal intensity (Carroll et al., 2003) and delay greater than the 98th percentile (Bhogal et al., 2022). To convert ${∆R}_{2}^{*}$ to concentration-time curve $C\left(t\right)$ in both blood and tissue voxels, this manuscript assumed a linear relationship $∆R_{2}^{*}\left(t\right)=r_{Y}\times C\left(t\right)$ , with coefficients $r_{Y\_tissue}=r_{Y\_blood}=1$ .

#### 2.4.3 Time delay (TD)

Since the signal at each voxel was a sinusoid, whose phase could be estimated, TD was computed as the phase delay of tissue:

$$TD=\frac{\phi_{tissue}-\phi_{venous}}{2\pi f_{c}}$$ (4)

where $f_{c}$ is the fundamental frequency of the sinusoidal stimulus and ϕ is the phase of the sine wave (Blockley et al., 2011) calculated from the Fourier transform of the BOLD signal with respect to time. The venous phase $\phi_{venous}$ was estimated by forming a histogram of phase delays across all voxels within the brain for each subject and selecting the 98th percentile, thus removing observer bias to obtain more consistent phase estimates (Bhogal et al., 2022).

#### 2.4.4 Cerebral blood flow (CBF)

For traditional DSC, CBF is usually calculated by deconvolution with singular value decomposition (SVD) between the tissue signal and the blood VOF signal: (Østergaard et al., 1996):

$$C_{tissue}\left(t\right)=C_{blood}\left(t\right)⊗\left(CBF\times R\left(t\right)\right)$$ (5)

For *SineCO*_*2*_, CBF was calculated in the frequency domain based on the magnitude spectra of Fourier-transformed tracer kinetics model (Eq. 4):

$$\left|C_{tissue}\left(f\right)\right|=\left|C_{blood}\left(f\right)\right|\times CBF\times \left|R\left(f\right)\right|$$ (6)

The residue function $R\left(t\right)$ was modeled using a decaying exponential function, $R\left(t\right)=e^{-t/\tau }$ with time constant τ (Østergaard, 2005). For a first-order system at low frequencies its time constant can be approximated as the phase delay (Thompson, 2014), τ=TD calculated from Eq. 4 The magnitude spectrum of the residue function was $\left|R\left(f\right)\right|=\frac{1}{\sqrt{{\left(1/\tau \right)}^{2}+{\left(2\pi f\right)}^{2}}}$ . CBF was the only unknown in Eq. 6 and was estimated using least-squares fitting for the magnitude spectra at each voxel.

#### 2.4.5 Cerebral blood volume (CBV)

CBV was calculated as $CBV=\frac{\kappa }{\rho }\frac{\int \left|C_{tissue}\left(t\right)dt\right|}{\int \left|C_{blood}\left(t\right)dt\right|}$ , where ρ is the brain density 1.05 g/mL, κ is the hematocrit correction factor, and the limits of integration represent the start and stop of the BOLD signal response. In respiratory-based DSC, since the deoxygenation contrast is confined within red blood cells instead of plasma, κ=1/0.69 is used to account for the difference between capillaries’ and large blood vessels’ hematocrits (Tudorica et al., 2002; Schulman et al., 2022).

#### 2.4.6 Mean transit time (MTT)

MTT was computed using central volume theorem as the ratio between CBV and CBF (Stewart, 1893):

$$MTT=\frac{CBV}{CBF}$$ (7)

#### 2.4.7 Cerebrovascular reactivity (CVR)

To generate the CVR maps, temporal alignment and least squares fitting were applied between the single-echo BOLD at TE of 35 ms and EtCO_2_ signals on a voxel-by-voxel basis, with the slope term as CVR calculation (Liu et al., 2019a). Maps of voxel-wise ratio between CBF and CVR for individual subjects were generated for regional comparison between the two parameters.

### 2.5 Statistical analysis

Statistical analysis was performed in R statistical package (R Core Team, Vienna, Austria). Amplitude, phase, and period of sinusoidal signals were computed by fitting the BOLD signals (either single-echo at TE of 35 ms or dual-echo at TEs of 35 and 90 ms) to sine waves while minimizing least-squares errors. Temporal SNR of the sinusoidal BOLD signal was calculated as the ratio between the peak-to-peak amplitude and the standard deviation of the BOLD fluctuations after removing the fundamental sine wave.

Perfusion measurements were checked for normality with Shapiro-Wilk test. To compare global perfusions between *SineCO*_*2*_ and DSC, ASL, or PC reference, paired *t*-test was performed. Reproducibility was assessed from two iterations of two-cycle sinusoid for *SineCO*_*2*_. Test-retest and intersubject coefficient-of-variation were reported.

Within each subject, agreement between pairs of methods was assessed using correlation and limits of agreement analyses. Pearson correlation coefficient r was calculated from the linear fit between perfusion values from 312 ROI for pairs of perfusion techniques. 95% limits of agreement were calculated as $\bar{d}\pm {1.96\times s}_{d}$ , where $\bar{d}$ is the mean difference and $s_{d}$ is the standard deviation of the differences between two methods in the ROI set. All limits of agreement were normalized by the average of the two methods and reported as percentages (Bland and Altman, 1999; Giavarina, 2015).

## 3 Results

### 3.1 Respiratory challenges

All 10 subjects successfully completed the *SineCO*_*2*_ respiratory challenge, but one subject was excluded from the group analysis because of gas leakage from the mask caused by facial hair, and one subject was excluded since the timing of the gas challenge and the BOLD imaging was incorrectly aligned at the time of the experiment.

None of the subjects reported discomfort during *SineCO*_*2*_ respiratory challenge. Baseline tidal volumes and respiration rates were 772 ± 292 mL and 17.2 ± 3.3 breaths/min and did not change significantly during CO_2_ modulations (*p* = 0.27 and *p* = 0.82, respectively). Initial EtCO_2_ and EtO_2_ recordings were 41.0 ± 3.5 mmHg and 110.4 ± 7.0 mmHg in the cohort. During *SineCO*_*2*_, continuous measurements of EtCO_2_ demonstrated sinusoidal amplitudes of 4.6 ± 0.8 mmHg (Figure 1A), whereas EtO_2_ was kept level at baseline (Figure 1B). SpO_2_ remained level at 98.4% ± 0.9% during the challenge.

Under CO_2_-induced vasodilation and vasoconstriction, single-echo gradient-echo MRI signal at TE of 35 ms varied in a sinusoidal pattern with peak-to-peak amplitude of 1.20% ± 0.44% (ΔR_2_^*^ = 0.34 ± 0.13 s^−1^) relative to baseline (Figure 1C), higher in the GM (1.52% ± 0.57%, ΔR_2_^*^ = 0.43 ± 0.16 s^−1^) compared to WM (0.58% ± 0.26%, ΔR_2_^*^ = 0.17 ± 0.07 s^−1^, *p*<0.01). Temporal SNR was 1.36 ± 0.52 in the whole brain, 1.72 ± 0.66 in GM, and 0.92 ± 0.38 in WM. In the Fourier domain, global signals demonstrated a peak at 0.17 Hz, corresponding to a sine wave period of 60 s (Figure 1D).

### 3.2 Perfusion measurements

#### 3.2.1 Single-echo *SineCO*_*2*_

**FIGURE 2:**
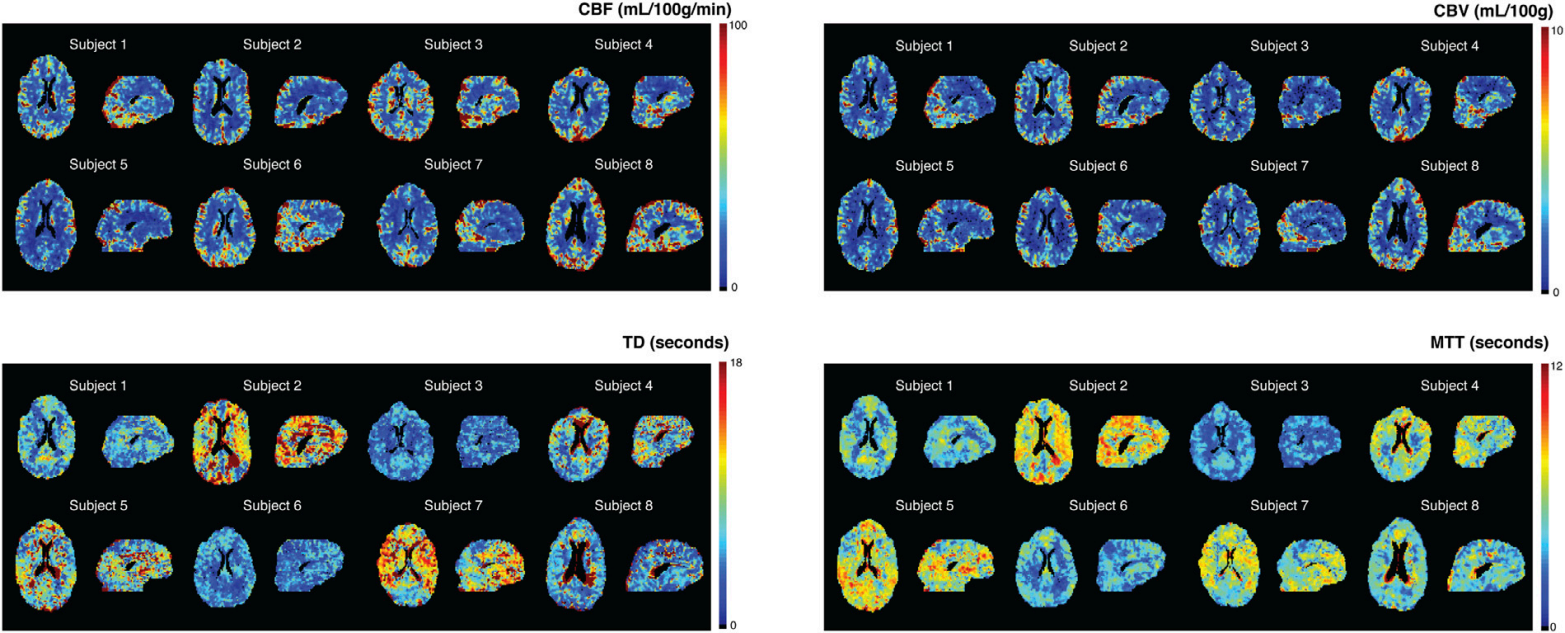
*SineCO*_*2*_ CBF, CBV, TD, and MTT maps for individual subjects.

Perfusion parameters for the whole brain, GM, and WM are displayed in Table 1; individual CBF, CBV, TD, and MTT maps by single-echo *SineCO*_*2*_ at TE of 35 ms are shown in Figure 2. Similar spatial distribution is observed in CBF and CBV maps (Figure 2), with GM-WM ratio of 2.1 ± 0.1 for CBV and 1.9 ± 0.1 for CBF. Both TD and MTT maps showed shorter venous delay in deep WM compared to GM (*p* = 0.02 and *p*<0.01, respectively), but distribution is heterogeneous between subjects (Figure 2).

**TABLE 1:** Regional perfusion estimates by SineCO_2_ and three reference standards ASL, DSC, and PC in the whole brain (WB), grey matter (GM), and white matter (WM).

		CBF (mL/100 g/min)	CBV (mL/100 g)	TD (seconds)	MTT (seconds)
*SineCO*_*2*_	WB	38.8 ± 7.5 (0.19)	3.1 ± 0.4 (0.11)	6.4 ± 2.2 (0.35)	4.5 ± 1.0 (0.23)
	GM	47.9 ± 9.2 (0.19)	3.9 ± 0.5 (0.12)	6.6 ± 2.2 (0.33)	4.8 ± 1.0 (0.20)
	WM	25.3 ± 5.7 (0.22)	1.7 ± 0.2 (0.11)	6.3 ± 2.2 (0.36)	4.1 ± 1.0 (0.25)
DSC	WB	29.6 ± 5.4 (0.18)	2.9 ± 0.3 (0.10)	2.4 ± 0.2 (0.10)	6.4 ± 1.0 (0.16)
	GM	35.6 ± 6.2 (0.17)	3.4 ± 0.3 (0.10)	2.1 ± 0.2 (0.11)	6.3 ± 0.9 (0.14)
	WM	20.3 ± 4.2 (0.21)	2.0 ± 0.2 (0.12)	2.6 ± 0.2 (0.08)	6.6 ± 1.3 (0.20)
ASL	WB	48.0 ± 7.8 (0.16)	NA	NA	1.13 ± 0.08 (0.7)
	GM	60.5 ± 10.8 (0.18)	NA	NA	1.07 ± 0.09 (0.08)
	WM	32.3 ± 8.0 (0.25)	NA	NA	1.22 ± 0.09 (0.07)
PC	WB	65.9 ± 8.3 (0.13)	NA	NA	NA

##### 3.2.1.1 CBF

To evaluate quantitative perfusion by *SineCO*_*2*_, mean CBF values are shown in Table 1 in comparison with gadolinium-based DSC, ASL, and PC. In the whole brain, *SineCO*_*2*_ CBF trended lower compared to ASL (*p* = 0.08) but was significantly lower than PC (*p* = 0.01) and higher than DSC (*p* = 0.04). In terms of reproducibility, there was no significant difference between two repetitions (*p* = 0.47) with a test-retest coefficient of variation of 21%. The intersubject coefficient of variation was 19%, slightly higher compared to DSC (18%), ASL (16%), and PC (13%).

**FIGURE 3:**
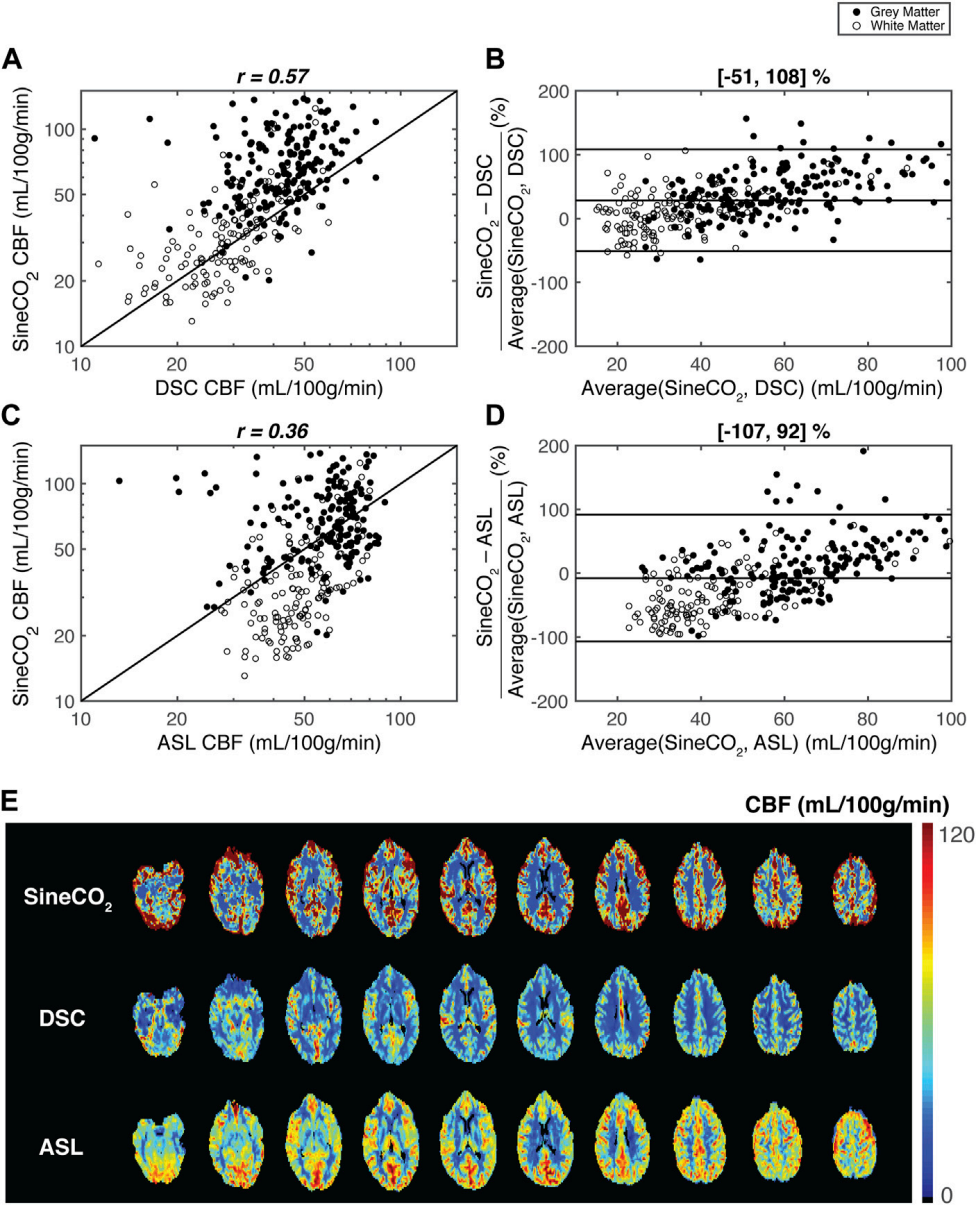
Regional agreement between respiratory challenge *SineCO*_*2*_ and reference standards DSC and ASL in a representation subject. Correlation and Bland-Altman limits of agreement analyses using 312 regions-of-interest between **(A–B)** *SineCO*_*2*_ and DSC and **(C–D)** *SineCO*_*2*_, and ASL **(E)** CBF maps in representative subject by three techniques.

Within-subject correlations and Bland-Altman analyses are shown for a representative subject in Figure 3, and individual analyses are in Supplementary Figures S1, S2, demonstrating similar correlation and width of the limits of agreement between *SineCO*_*2*_ and reference techniques compared to agreement amongst DSC and ASL references.

##### 3.2.1.2 CBV

*SineCO*_*2*_ was not different from CBV by DSC (*p* = 0.36), and regional correlation was high across ROIs and similar to agreement observed in CBF (Supplementary Figure S3). Intersubject coefficient of variation was 11%, and test-retest coefficient of variation was 20%, demonstrating no significant difference between the two repetitions (*p* = 0.18).

##### 3.2.1.3 TD

TD values by *SineCO*_*2*_ were significantly longer compared to DSC (*p* < 0.01). Opposite trends were observed between the two techniques, with prolonged TD in WM in DSC but shortened WM delay relative to venous signal in *SineCO*_*2*_ challenge. Compared to CBF and CBV measurements, TD maps were noisier (Figure 2C), with a test-retest coefficient of variation of 25% and intersubject coefficient of variation of 35%.

##### 3.2.1.4 MTT

Shorter MTT values were observed in *SineCO*_*2*_ compared to DSC (*p* = 0.01), and grey matter showed longer transit time than white matter (*p* < 0.01). No correlation was observed with DSC MTT (not shown). Test-retest and intersubject coefficients of variation were 17% and 23%, respectively.

#### 3.2.2 Dual-echo *SineCO*_*2*_

Compared to the global ΔR_2_^*^ 0.34 ± 0.13 s^−1^ obtained at the first TE = 35 ms, dual-echo ΔR_2_^*^ at TEs of 35 and 90 ms was 0.22 ± 0.08 s^−1^ (*p* < 0.01). Temporal SNR was lower in the dual-echo signal (tSNR = 0.82 ± 0.32, *p* = 0.03). Individual CBF, CBV, TD, and MTT maps using the dual-echo approach (Supplementary Figure S4) show a similar spatial distribution compared to single-echo perfusion maps (Figure 2). However, dual-echo maps are noisier and yield a higher bias in CBF compared to DSC and ASL (data not shown).

### 3.3 Cerebrovascular reactivity

**FIGURE 4:**
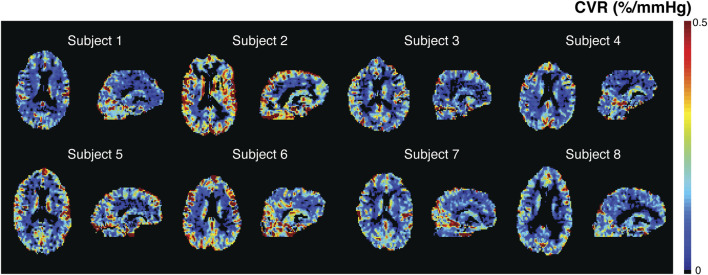
*SineCO*_*2*_ CVR maps in individual subjects.

Individual CVR maps are shown in Figure 4. Mean CVR was 0.24% ± 0.06%/mmHg in the cohort, significantly higher in the GM (0.28% ± 0.07%/mmHg) compared to the WM (0.13% ± 0.03%/mmHg, *p*<0.01). Spatial patterns of CVR maps are similar to CBF and CBV maps generated from the *SineCO*_*2*_ technique. Ratio maps between CBF and CVR (Supplementary Figure S5) demonstrated areas of negative CVR in the deep white matter areas as well as disproportionally higher ratio in the white matter compared to GM.

## 4 Discussion

In this work, we employed a technique previously used to measure CVR with a CO_2_ respiratory challenge to modulate cerebral saturation and BOLD signal in a sinusoidal pattern (Blockley et al., 2011), after which tracer kinetics equations were applied in the frequency domain to compute perfusion parameters. *SineCO*_*2*_ CBF, and CBV values were within acceptable range of literature (Grandin et al., 2005), but MTT was larger than expected (Ibaraki et al., 2007). Single-echo acquisition yielded better temporal SNR and better image quality compared to dual-echo approach. CBF estimates were compared with three reference techniques, gadolinium-based DSC, ASL, and PC, and demonstrated no bias with ASL and PC but overestimation compared to DSC. The limits of agreement were large between *SineCO*_*2*_ with ASL, and DSC but were comparable to agreement amongst the reference techniques and previously reported agreement between DSC and PET (Grandin et al., 2005). Despite the systematic biases, perfusion maps showed regional agreement between the techniques, indicating that *SineCO*_*2*_ has the potential to differentiate diseased and normal-appearing tissue in cerebral pathologies such as ischemic strokes or brain tumors.

The use of CO_2_ vasoactive stimulus represents a divergence from previous deoxygenation-based DSC studies, which utilize hypoxia or hyperoxia respiratory challenges to directly deliver boluses of deoxygenated hemoglobin (MacDonald et al., 2018; Poublanc et al., 2021; Vu et al., 2021). Capnic challenges raise and lower cerebral saturation through vasodilation and vasoconstriction within the capillary beds, so the sinusoidal modulations are not present on the arterial side but instead only in vessels undergoing oxygen exchange and large veins. Therefore, this source of contrast results in an anti-causal system where the VOF is used *in lieu* of an AIF. Conceptually, this is analogous to playing a cine-angiogram in reverse. Even though the anti-causality is not compatible with traditional tracer kinetics model (Meier and Zierler, 1954; Østergaard, 2005), computation of CBF in the frequency domain ignores the phase in favor of the magnitude, which is independent of the relative delay between tissue and VOF signals. CBV estimates in typical DSC experiments are corrected with the area-under-curve of a VOF signal, which is usually less vulnerable to partial volume effects compared to AIF (Knutsson et al., 2010); therefore, CBV measures are also independent of the use of VOF. On the other hand, TD is calculated as the delay between the phase of the tissue signal and venous phase for *SineCO*_*2*_ instead of arterial phase in DSC; therefore, TD maps demonstrated opposite trends between the two techniques.

The *SineCO*_*2*_ approach has some interesting properties. Overall, since the endogenous contrast is generated by oxygen exchange, it cannot detect actual or effective shunt flow, potentially underestimating true perfusion. The contrast change results from a cascaded transport system, in which the CO_2_ stimulus passes through an initial cerebrovascular response transfer function followed by a secondary residue function that governs the propagation of deoxyhemoglobin. The complexity of this higher-order system is simplified by the capability to extract a VOF signal, which relates to the signal only through the residue transfer function. The indirect mode of contrast generation also requires some cerebrovascular reactivity to generate a signal suitable for CBF estimation, hence it is not surprising that CVR, CBF, and CBV maps resemble one another. In brain regions where resting flow is preserved but CVR is abnormal, signal-to-noise of the CBF and CBV estimates will be poor.

CO_2_ modulations also have complex cerebrovascular and peripheral hemodynamic effects, including changes in respiratory rates, tidal volumes, heart rates, blood pressures, and perfusion values, proportionally to the extent and duration of CO_2_ inhalation. The upward swing of the CO_2_ sinusoid is a hypercapnic stimulus which results in increase in CBF (Kety and Schmidt, 1948), whereas the trough of the sinusoid represents a hypocapnic stimulus with a decrease in flow. Assuming ±5 mmHg fluctuations in EtCO_2_ remain within the autoregulatory range (Battisti-Charbonney et al., 2011), 1 mmHg change in EtCO_2_ typically induces 1–2 mL/100 g/min change in CBF (Kety and Schmidt, 1948; Brian, 1998). Therefore, CBF in the tracer kinetic model can be written as a function of time $CBF\left(t\right)={CBF}_{0}\left[1+CVR\left({EtCO}_{2}\left(t\right)-40\;mmHg\right)\right]$ , where ${CBF}_{0}$ is the baseline cerebral blood flow at EtCO_2_ of 40 mmHg. With a typical grey matter CVR of 0.2%/mmHg, and a peak-to-peak amplitude of 5 mmHg, the oscillating contribution is only around 1%. Furthermore, since the oscillations are centered about the most linear portion of the CBF–EtCO_2_ curve (Battisti-Charbonney et al., 2011), this work assumes that the value measured is the average perfusion and is comparable to baseline blood flow. However, this assumption requires validation with a dynamic acquisition of ASL or PC with high enough temporal resolution to quantify fluctuations in CBF in response to CO_2_ respiratory challenge.

Additionally, the vasoactive effects of CO_2_ challenge can potentially explain the divergence in regional agreement between *SineCO*_*2*_ and ASL. During sustained hyperemia, cortical GM regions are prioritized compared to deep WM (Chai et al., 2019). This steal phenomenon occurs in which blood flow preferentially increases in GM at the expense of WM (Poublanc et al., 2013), causing higher sinusoid amplitudes in GM and thus overestimation of CBF in cortical regions. On the other hand, since flow changes are lower within WM, CBF measurements are not as high in WM in *SineCO*_*2*_ compared to baseline measurements by ASL.

Other limitations include the study design of administering the sinusoidal stimulus about a fixed EtCO_2_ value of 40 mmHg regardless of the subject’s initial EtCO_2_ levels; in subjects of high baseline CO_2_ partial pressure, this paradigm induced hypocapnia and hyperventilation response that lengthened transit time (Ito et al., 2003) and potentially explained the heterogeneous distribution in several TD maps (Angleys et al., 2015). Clamping the average end-tidal CO_2_ to 40 mmHg may also introduce a small “step” response in the BOLD signal for individuals whose resting end-tidal CO_2_ is far from 40 mmHg. However, limiting our analysis to a single frequency minimized error contributions from this effect. Despite targeting a single fundamental frequency *f*_*c*_, in practice only a perfect sinusoid can be accomplished on positive cycles. The shape of the negative cycle depends on the subject’s hyperpneic response from the previous positive cycle, thus introducing a small non-linearity and frequencies outside the target range. Lastly, since the signal is venous-weighted, SpO_2_ values could not be used to convert $∆R_{2}^{*}$ to arterial saturation in concentration-time curves; therefore, the values presented here are only considered semi-quantitative. However, since relative perfusion is frequently used in clinical routines, semi-quantitative measurements may still offer insight into diseased tissue relative to contralateral normal-appearing tissue.

Despite the shortcomings, the most significant advantage to *SineCO*_*2*_ perfusion imaging is that sinusoidal CO_2_ respiratory challenge is a robust mechanism to measure CVR (Blockley et al., 2011). Previous works have demonstrated that 32% of the variation in GM CVR is explained by variation in baseline CBF (Afzali-Hashemi et al., 2021), so these two parameters are tightly coupled together and are known to vary with changes in EtCO_2_ (Hou et al., 2020). However, measurement of CVR can still yield additional information, as illustrated by the existence of negative CVR values in deep WM unseen on CBF maps. Divergence in CBF and CVR as shown in the ratio maps typically happens in areas of low flow and long delay, in which CBF can increase in response to CO_2_ but requires sufficient time to reach the hypercapnic ceiling and can potentially be classified as negative CVR (Poublanc et al., 2015). In this current technique, *SineCO*_*2*_ CBF measurements are calculated purely from the magnitude spectrum and are independent of phase delay, but CVR estimates computed from traditional general linear model approach are influenced by vascular delay (Poublanc et al., 2015; Liu et al., 2019b). Therefore, *SineCO*_*2*_ capability to acquire both perfusion and reactivity simultaneously in one imaging sequence is of high interest in cerebrovascular diseases and gives it an edge over other conventional perfusion MRI techniques.

Most of *SineCO*_*2*_ potential diagnostic power lies in perfusion imaging of strokes or gliomas, especially in more vulnerable populations in whom gadolinium injection is undesirable, such as renal-impaired or pediatric patients. However, the fundamental difference between gadolinium contrast and deoxyhemoglobin contrast may allow them to play complementary roles in perfusion imaging for these pathologies. For ischemic strokes in which the penumbra is under low oxygen delivery, CO_2_-induced modulations in CBF can lead to reperfusion of the damaged regions (Brambrink and Orfanakis, 2010), which can yield a completely different perfusion distribution compared to gadolinium DSC. In brain tumors, gadolinium-based contrast extravasation through the disrupted blood-brain barrier can result in altered CBV measurements (Ho et al., 2016); on the other hand, deoxygenation-based contrast remains purely intravascular. Therefore, CBV measured using gadolinium-based DSC within gliomas might differ compared to *SineCO*_*2*_ CBV. These potential divergences in the two techniques require additional work to evaluate the diagnostic role of *SineCO*_*2*_ in different cerebrovascular pathologies.

In conclusion, this validation study established feasibility of using *SineCO*_*2*_ to measure perfusion and demonstrated agreement between *SineCO*_*2*_ against three reference perfusion techniques, DSC, ASL, and PC. Despite the systematic bias, in clinical routines, neuroradiologists typically rely on relative perfusion differences between diseased and normal-appearing tissue rather than absolute perfusion, so *SineCO*_*2*_ relative perfusion maps may still be useful clinically independent of VOF selection. Additionally, *SineCO*_*2*_ also represents an easy approach to generate CBF maps independent of confounding parameters in SVD deconvolution and minimize MRI time by simultaneous acquisition of perfusion and reactivity in one imaging sequence.

## Data Availability

De-identified imaging data and processing code will be made available to qualified researchers on a case-by-case basis after obtaining approval from the Children’s Hospital Los Angeles regulatory authorities.
