# Supplementary material for: Sinusoidal CO2 respiratory challenge for concurrent perfusion and cerebrovascular reactivity MRI
Source: Front Physiol. 2023 Feb 9;14:1102983. doi: 10.3389/fphys.2023.1102983 (PMC9948030; doi:10.3389/fphys.2023.1102983)
Supplement: Supplementary file 1 [file Table1.DOCX]

Sinusoidal CO_2_ respiratory challenge for concurrent perfusion and cerebrovascular reactivity MRI

Supplementary Material

**Supplemental Figures:**


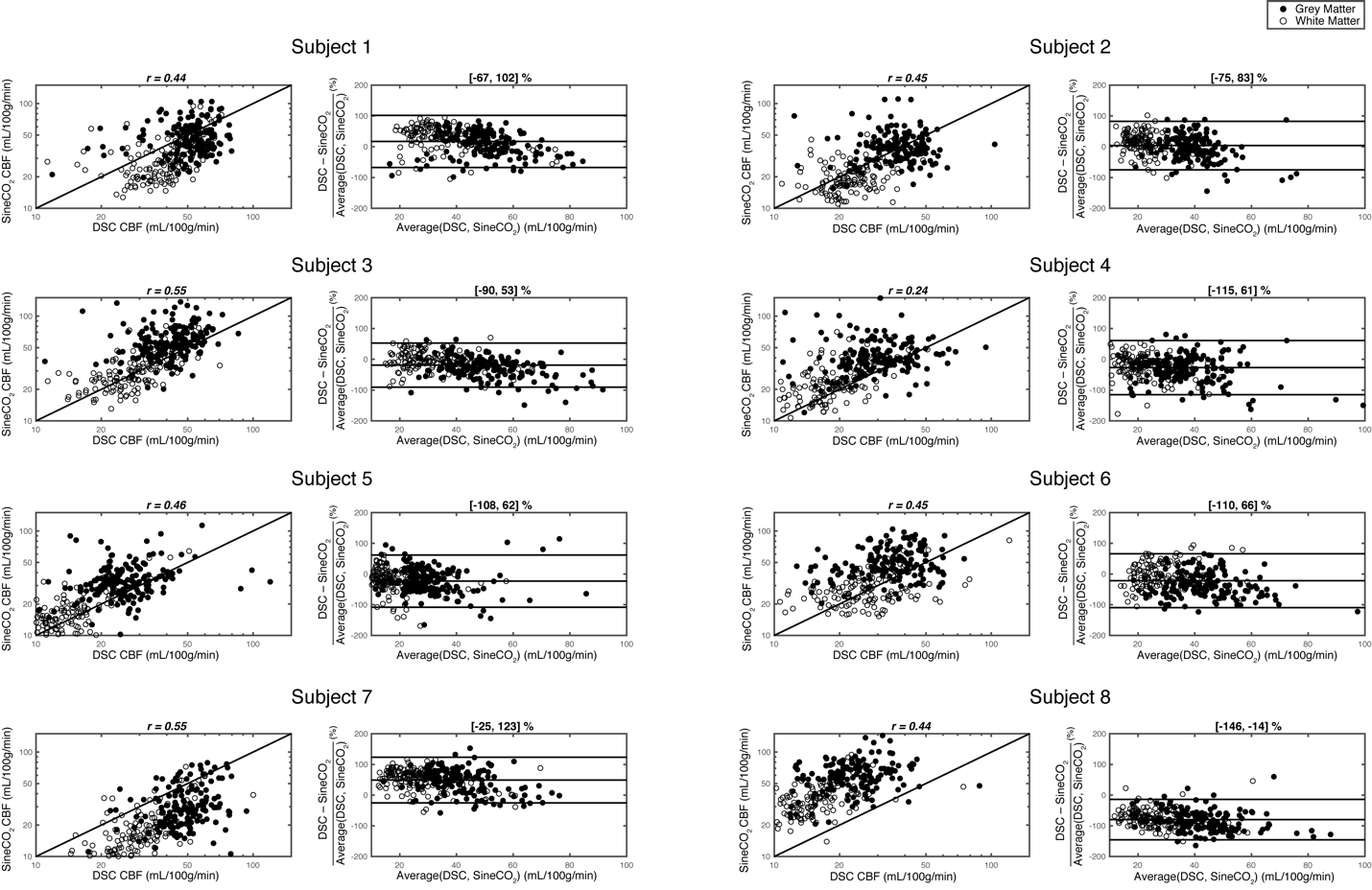


Supplemental Figure S1. Regional agreement in CBF between *SineCO_2_* and DSC using 312 regions-of-interest.


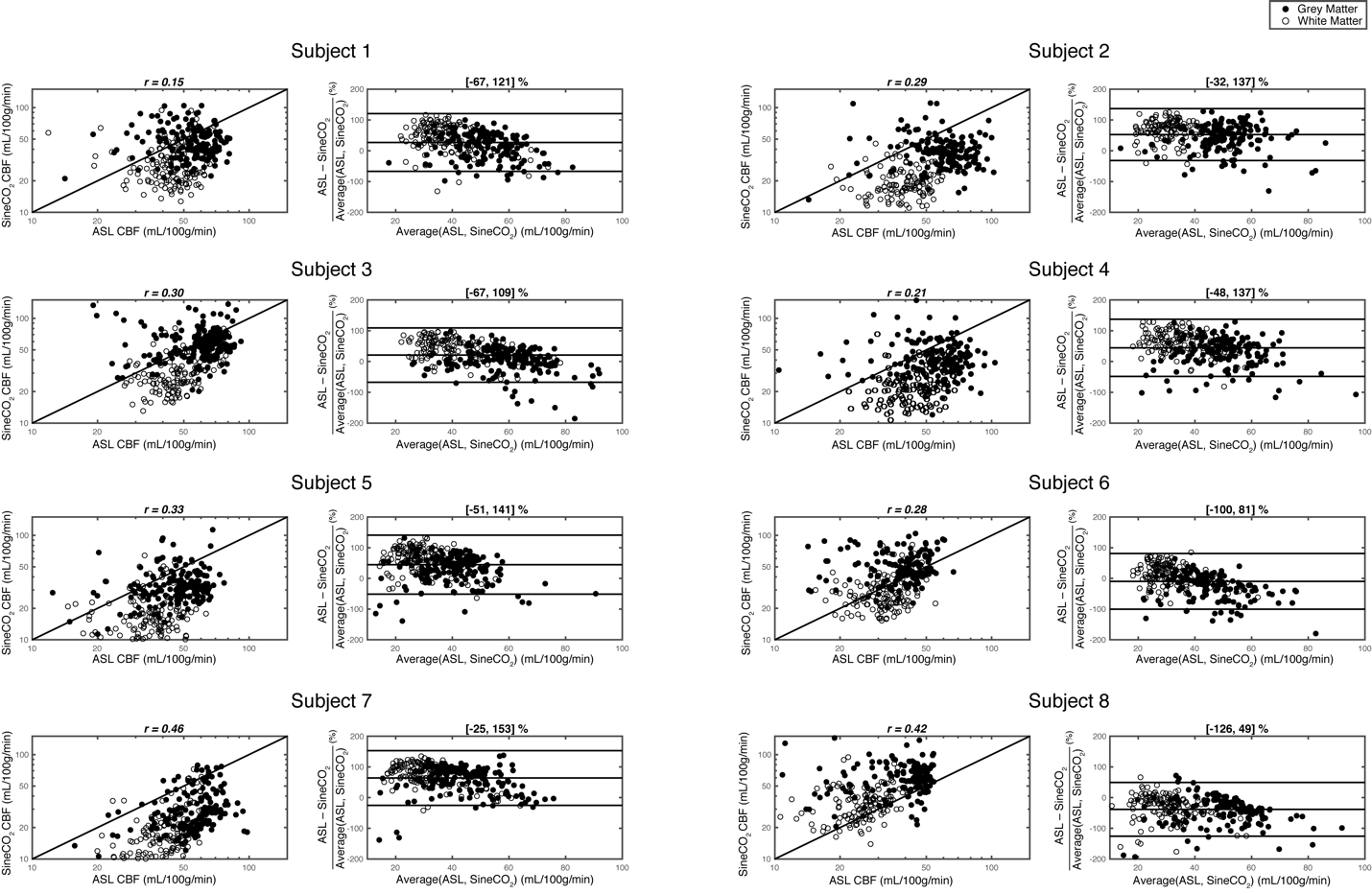


Supplemental Figure S2. Regional agreement in CBF between *SineCO_2_* and ASL using 312 regions-of-interest.


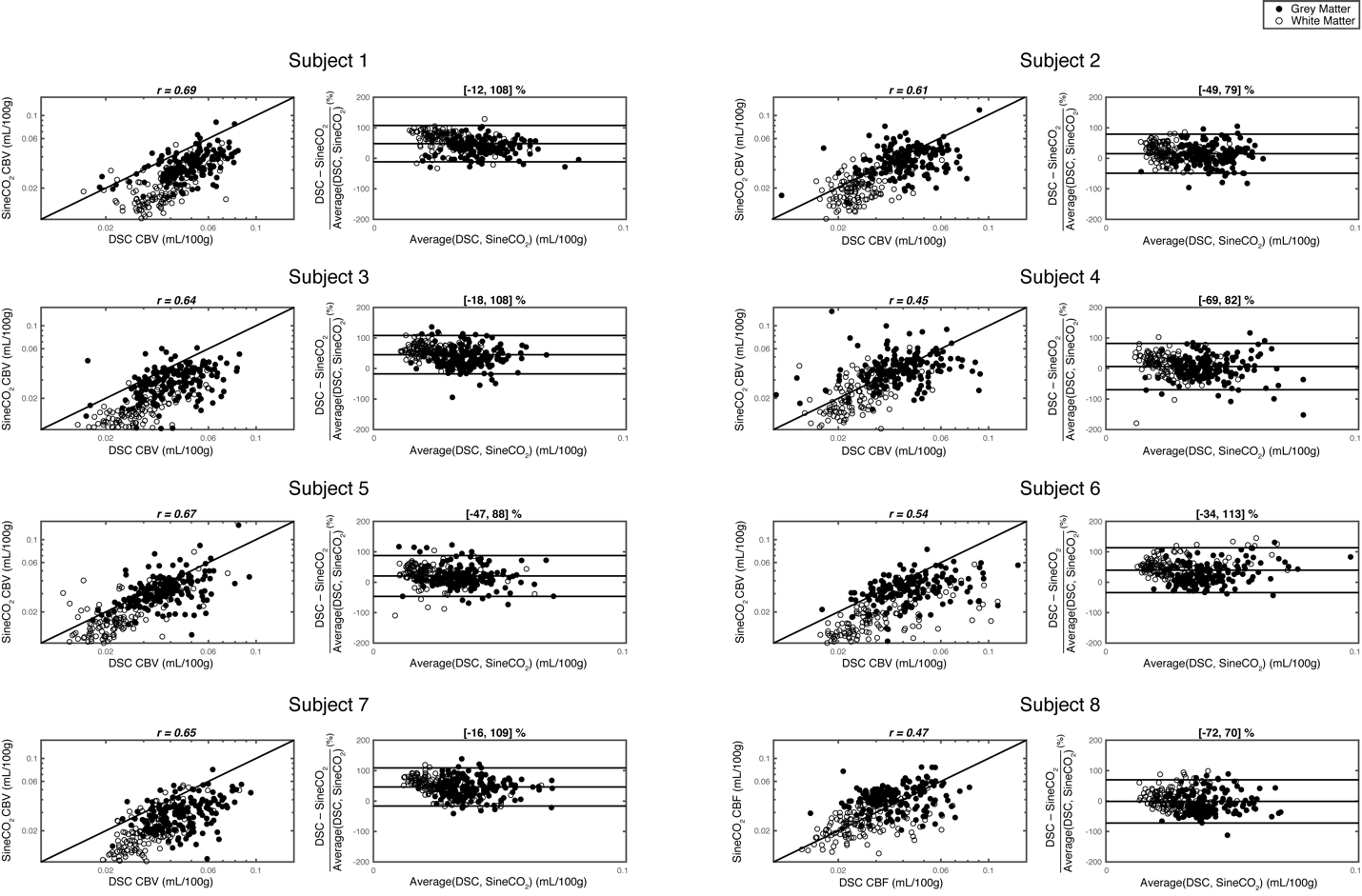


Supplemental Figure S3. Regional agreement in CBV between *SineCO_2_* and DSC using 312 regions-of-interest.


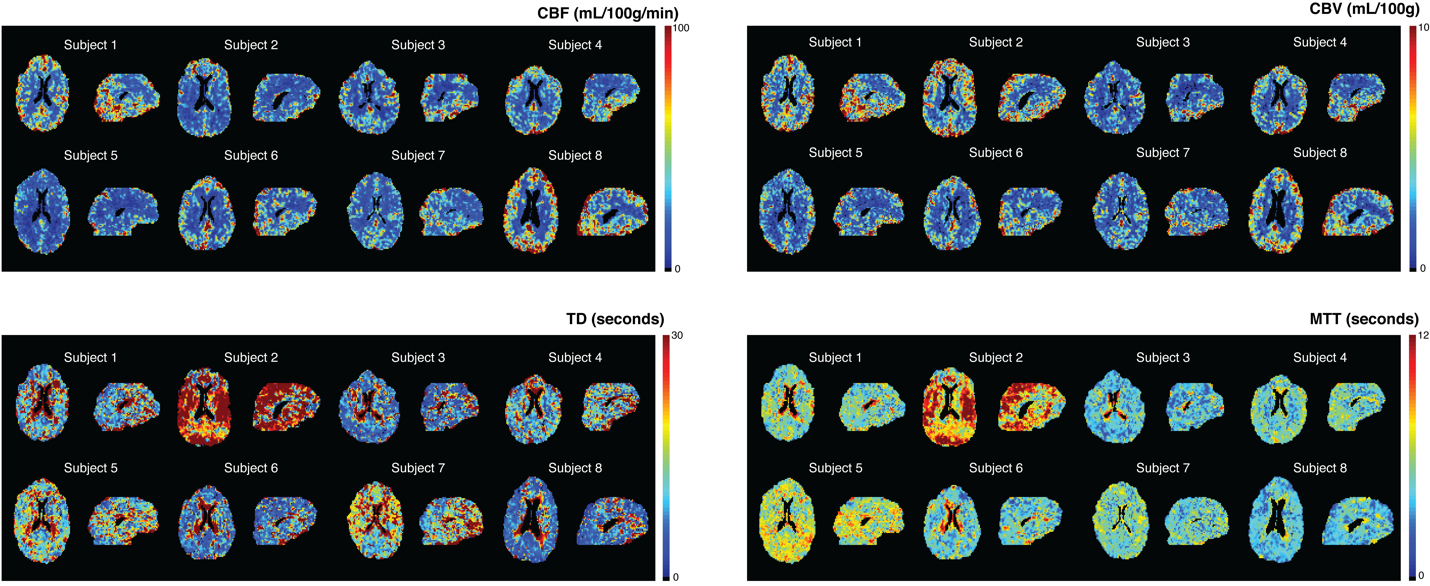


Supplemental Figure S4. *SineCO_2_* dual-echo CBF, CBV, TD, and MTT maps for individual subjects.


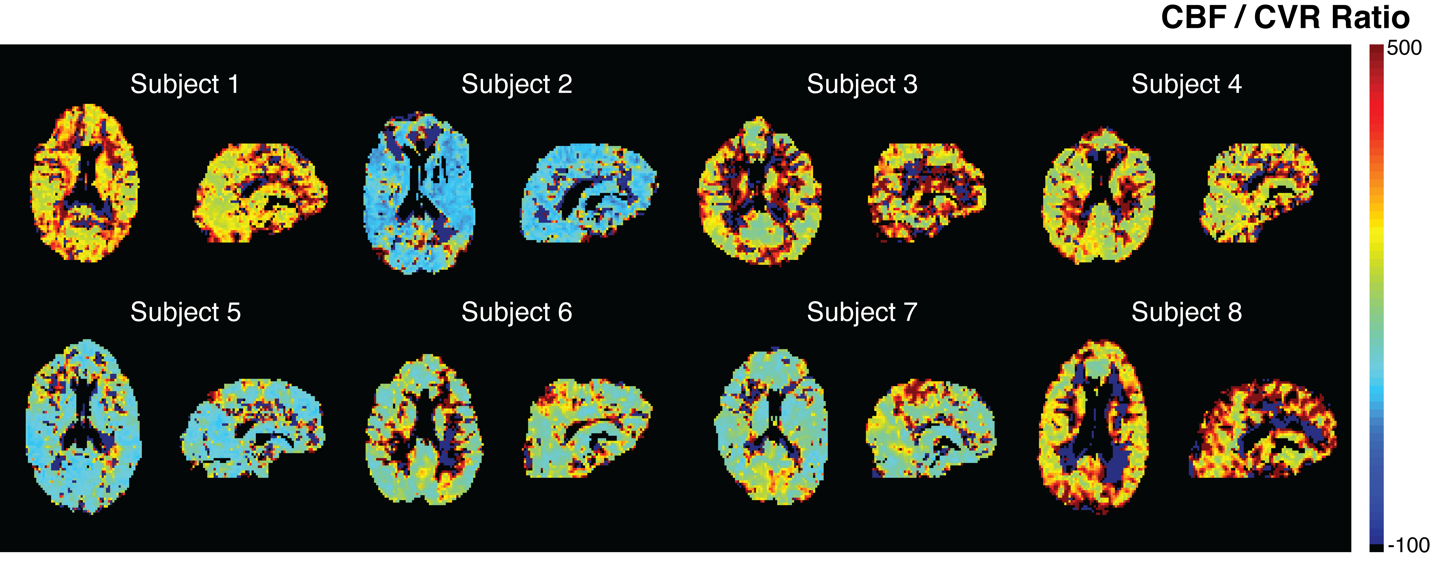


Supplemental Figure S5. Maps of CBF and CVR ratio in individual subjects.
